# Time to death and its predictors among neonates who were admitted to the neonatal intensive care unit at tertiary hospital, Addis Ababa, Ethiopia: Retrospective follow up study

**DOI:** 10.3389/fped.2022.913583

**Published:** 2022-08-29

**Authors:** Mulat Mossie Menalu, Bereket Gebremichael, Kalkidan Wondwossen Desta, Worku Misganaw Kebede, Fetene Nigussie Tarekegn, Getaneh Baye Mulu, Bantalem Tilaye Atinafu

**Affiliations:** ^1^School of Nursing and Midwifery, Asrat Weldeyes Health Science, Debre Berhan University, Debre Berhan, Ethiopia; ^2^College of Health Sciences, Addis Ababa University, Addis Ababa, Ethiopia; ^3^Department of Pediatrics and Child Health Nursing, School of Nursing and Midwifery, Institute of Medicine and Health Science, Addis Ababa University, Addis Ababa, Ethiopia; ^4^Clinical and Pyschosocial Epidemiology, Faculty of Medical Sciences, University of Groningen, Groningen, Netherlands

**Keywords:** neonatal mortality, survival status, time to death, predictors, Ethiopia

## Abstract

**Backgrounds:**

Neonatal death is the major problem in developing world. Burden and predictors of neonatal mortality vary across countries and even among regions of a country, so understanding the problem concerning these factors is essential to overcome the problem. Therefore, this study aimed to determine time to death and its predictors of neonatal mortality among neonates who were admitted to the neonatal intensive care unit of Tertiary Hospital, Addis Ababa, Ethiopia.

**Methods:**

A hospital-based retrospective cohort study was employed among 434 neonates admitted in Tertiary hospital, Addis Ababa, Ethiopia. A Kaplan Meier curve and a log-rank test were used to estimate the survival time and compare survival curves between variables. The cox proportional hazard model was also fitted to identify predictors.

**Results:**

A total of 434 neonates included in the study, 11.1% of which were died, and the incidence rate was 19.2 per 1000 live births. The time to death of neonates was 17 days. Independent predictors of neonatal mortality were incomplete maternal antenatal follow up[AHR: 3.7 (95% CI:1.86,7.60)], low(Appearance, Pulse, Grimily, Activity, and Respiration(APGAR)score[AHR:5.0 (95%CI:1.51–15.04)], perinatal asphyxia [AHR:5.2 (95%CI:1.92–14.30)], preterm 4.2 (95%CI: 1.32–8.83)]. Moreover, small for gestational age [AHR:4.8 (95%CI:2.33–9.72)], respiratory distress[AHR: 2.5 (95%CI: 1.24–5.09)], sepsis [AHR: 3.4 (95%CI: 1.71–4.01)], low birth weight[AHR: 7.3 (95%CI:2.69,1.91)], and tracheoesophageal fistula [AHR: 2.2 (95%CI: 1.13–4.32)].

**Conclusion:**

The overall incidence rate was 19.2 deaths per 1,000 live births. Emphasis should be given to incomplete Antenatal care follow up, small for gestation, preterm, low birth weight, low 5^th^ min APGAR score, neonatal sepsis, respiratory distress, perinatal asphyxia, and tracheoesophageal fistula.

## Introduction

Globally, it is estimated that ~6,500 neonatal deaths occurred every day. Of those, about a third of all neonatal deaths occurred within the first day after birth, and three-quarters occurring within the first week of life (1). Besides, the neonatal mortality rate was 37% in 1990, 31% in 2000 and 18% in 2017 (2). Despite, there is a decline in neonatal mortality from 1990 to 2017, it is slower than post-neonatal and under-5 mortalities (2–5). Neonatal death is unevenly distributed based on age, socio-demographic population groups, and other factors (2, 6). The 2022 global report bared that the highest neonatal mortality reported in Sub-Saharan Countries which was 27 deaths per 1,000 live births with 43% of global newborn deaths, followed by central and southern Asia which was 23 deaths per 1,000 live births, with 36% of global newborn deaths. Moreover, a child born from Sub-Saharan Countries is 10 times more likely to die than child born from high income countries (7).

The three major causes of neonatal mortality in developing countries include prematurity, infections, and perinatal asphyxia (2, 8, 9). Newborns that need intensive medical attention are often admitted into a special area of the hospital called the neonatal intensive care unit [NICU]. A new initiative called the Global Strategy for Women's, Children's, and Adolescent's Health [2015–2030] and the third Sustainable Development Goal [SDG] has been established to ensure healthy lives and promote wellbeing for all ages (2). The SDG goal 3 geared toward preventing deaths of newborns specifies that all countries should aim to reduce neonatal mortality to at least as low as 12 deaths per 1,000 live births by 2030, but without strengthened commitment to newborn survival, many countries couldn‘t meet the SDG goal to end preventable neonatal deaths (2).

In Ethiopia, a 2020 report estimated that neonatal mortality rate for Ethiopia was 27 deaths per 1,000 live births. The rate of neonatal mortality decreased from 47.8 deaths per 1,000 live births to 27 deaths per 1,000 live births in 2001 and 2020, respectively (10). Over two-thirds of neonatal deaths are mainly due to infections and neonatal medical conditions (11–13). The neonatal causes of death are prematurity [21.8 %], infections [18.5 %], asphyxia [31.6 %] which together account for nearly 70% of deaths in this age group (14).

In particular, under-five mortality rates declined from 166 to 67 deaths per 1,000 live births in 2016. Similarly, infant mortality decreased from 97 to 48 deaths per 1,000 live births in the same period, but the neonatal mortality decreased from 54 to 29 per 1,000 live births (14–17). This indicates the trend of neonatal mortality reduction in Ethiopia is moving at a slow step compared to the reduction of infant and under-five mortality over the same period. National analysis data showed that multiple birth neonates, neonates born to mothers who did not utilize ANC and neonates from rural area were predictors of neonatal mortality (18).

Despite different initiatives and implementations that have been implemented to prevent neonatal death, it is still high and not reduced as expected in developing countries including Ethiopia. Although several studies were made in different countries on the survival status and predictors of mortality among neonatal intensive care unit [NICU] admitted neonates, but only a few studies were done in Ethiopia. Moreover, studies regarding neonatal mortality have focused on rate and little has been done on the time to death and predictors.

Therefore, this study aimed to determine the time to death and its predictors of neonatal mortality among neonatal intensive care unit admitted neonates at Tertiary Hospital Addis Ababa, Ethiopia.

## Methods

### Study setting, period, design

A retrospective cohort study was conducted at tertiary hospital Addis Ababa Ethiopia among live-born neonates who were admitted to NICU from 1 January 2015 to 30 December 2018. Addis Ababa has ten sub-cities at which the City lies at an altitude of 7,546 feet [2,300 meters] (19). There are three referral hospitals in Addis Ababa, Tikur Anbessa Specialized Hospital, Pawlos Hospital and Yekatit 12 Hospital, among this Tikur Anbessa Specialized Hospital (TASH) was randomly selected. TASH is a country's largest and iconic referral hospital for Ethiopia. The Tikur Anbessa Specialized Hospital NICU ward can accommodate a maximum of 60 patients with an average of 20–40 patients daily admission (20, 21).

### Eligibility criteria

All live-born neonates who were admitted to NICU of Tikur Anbessa Specialized Hospital from 1 January 2015 to 30 December 2018, Addis Ababa, Ethiopia were included. Whereas, Neonates of incompletely registered NICU logbooks and lost records at the time of data reviewing were excluded from the study.

### Sample size determination and sampling procedure

The sample size was determined by using double population proportion in a STATA statistical program considering the following assumptions: 95% CI, power 80%, *P1* = 10 % *P2* = 20% (22), and 10 % incomplete data, yielding a total sample size of 434.

A sampling frame was prepared from the medical record of neonates that were admitted in NICU from 1 January 2015 – 30 December 2018 [*N* = 12,888], then for each year, proportional allocation was taken, Randomly, the 2^nd^ MRN was selected in the same fashion for the rest 3 years, and then systematic sampling was employed every 30^th^ (Supplementary 1).

### Study variables

Time to death was an outcome variable. Independent variables includes:-socio-demographic factors (maternal age, neonatal age, and sex); maternal health factors [(Antenatal care) ANC follow up, RH (Rhesus) factor, blood group; maternal medical disorders like hypertension, diabetic mellitus, HIV/AIDS, and others, and gynecologic-obstetric related factors like gravidity, parity, mode of delivery, multiple pregnancies, premature rupture of membrane, abortion, prolonged labor); neonatal factors (Age at admission, sex, the weight of neonate, date of admission and discharge. APGAR score, gestational age, maturity, neonatal medical conditions: asphyxia, congenital malformation, preterm, sepsis, hypothermia, RDS (respiratory distress syndrome), MAS(Meconium aspiration syndrome), HMD (Hyaline membrane disease), jaundice, and TEF (trachoesophageal fistula)]; health service-related factors (duration of hospital stay, access to emergency obstetric care, resuscitation efforts).

### Operational definitions

Censored: didn't know survival time exactly due to study ends, loss to follow-up, withdrawal or being survived from study.

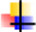
 Low APGAR score: a score < 7.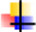 Event: death after NICU admission.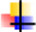 Asphyxia: a neonate with an APGAR score of <7.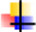 Time to death: it is the time from admission at NICU to the occurrence of the outcome/event.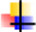 Time scale: days from admission of a neonate to the occurrence of an outcome.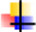 Time origin: date of admission or start time of the cohort.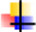 Complete ANC follow up: ANC visit≥4 times at current pregnancy period.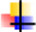 Multigravida: recorded gravida of ≥2.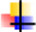 Multiparity**:** recorded parity of ≥2.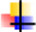 Preterm birth: any infant born before 37 weeks of gestational age.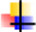 Low birth weight (LBW): birth weight <2500 gm.

### Data collection tools and procedures

Data was collected by using a checklist developed from neonatal intensive care unit registration book format, which was prepared nationally as an "Ethiopian NICU form. The checklist consisted of new-born information recorded at admission and records of maternal information which were collected using a uniform extraction format developed by taking in to account all the relevant variables in the standard NICU registration book. Then all medical records that fulfill the inclusion criteria were reviewed retrospectively.

The checklist was prepared in English. A pretest was done on 5% of the sample size in Yekatit 12 hospital before the time of the actual data collection. The checklist was also examined by experts to check for content validity.

The data reviewers and supervisors were 8 BSc and 3 MSc Nurses. Before starting the actual work, one-day training and orientation were provided to chart reviewers on the objectives of the study, data reviewing techniques, how to keep confidentiality of information, how to fill the data abstraction checklist and data quality management. Close supervision was carried out by supervisors and investigators to ensure data quality. Then, the collected data was checked for its completeness and consistency before analysis.

### Data processing and analysis

Data was coded, cleaned, edited and entered using Epi-data version 4.21 and exported to STATA SE version 14 for analysis. The event of interest was death. The time to death was calculated in days between the date of admission and the date of death. The log-rank test employed to illustrate whether considered covariates in the Kaplan Meier curves are statistically significant and in line with the Kaplan Meier curves. The bivariable cox regression was done and those variables having *p*-value < 0.25 in the bivariable analysis were included in the multivariable cox-proportional hazards regression model. Then, those variables with *P*-value ≤ 0.05 at 95% confidence interval were considered as statistically significant.

Kaplan Meier survival curve together with log-rank test was fitted to test the survival time of neonates, and their survival curves didn‘t cross over each other. A goodness-of-fit (23) test was employed to assess the proportional hazard [PH] assumptions for each predictor variable. The result showed that variables included in the model satisfied the proportional hazard assumption [*p*-value was 0.7156 > 0.05], which indicates that the PH assumption has been met.

## Results

### Socio-demographic characteristics of mother and neonate

About 434 charts were reviewed with a response rate of 100%. Around 54 [12.4%] of mothers were below age twenty, 262 [61%] between 20–34 years old, 114 [26.5] between 35–45, and 4 [0.04%] were above 45.

Among 239 males 27 [11.3%] of neonates died, and among 195 females 21[10.8%] of them died. The pattern of neonatal death in the 1st 24 h,1st 7 days, within the first 14 days, and the 1st 28 days was 12.9,7.6,11.1, and18.2%, respectively (Table 1).

**Table 1:** Socio-demographic characteristics of neonate and mother in NICU, TASH, Addis Ababa, Ethiopia, from 1 January 2015 – 30 December 2021.

**Variables**	**Category**	**Outcome [*****N*** = **434]**		**Row total**
		**Death**	**Censored**	
		**Count [%]**	**Count [%]**	**Count [%]**
Sex of a neonate	Male	27 [11.3]	212 [88.7]	239 [55%]
	Female	21 [10.8]	174 [89.2]	195 [45%]
Maternal age	≤ 20	4 [7.4]	50 [92.6]	54 [12.4%]
	20–34	34 [13.0]	228 [87.0]	262 [61%]
	35–45	10 [8.8]	104 [91.2]	114 [26.5]
	≥45	0 [0.0]	4 [100.0]	4 [0.04%]
Neonatal age	0–1day	21 [12.9]	142 [87.1]	163 [37.6]
	1–7days	12 [7.6]	145 [92.4]	157 [36.1]
	7–14days	9 [11.1]	72 [88.9]	81 [18.7]
	14–28days	6 [18.2]	27 [81.8]	33 [7.6]

### Maternal health conditions

Neonates born from mothers who had a complete antenatal follow up during their current.

Pregnancy, their death was 48/1,000 neonates, but neonates born from mothers of incomplete ANC was 360/1,000 neonates, and 615/1,000 for neonates born from mothers who did not have antenatal follow up were died.

About 24 [11.9%] of neonates born from primigravida mothers, 201 [46.3%] were died, and 24 [10.3%] of neonatal death was from multi gravid mothers 233 [53.7%] (Table 2).

**Table 2:** Maternal health conditions in NICU, TASH, Addis Ababa, Ethiopia, from 1 January 2015 – 30 December 2021.

**Variables**	**Category**	**Outcome [*****N*** = **434]**		**Row total**
		**Death**	**Censored**	
		**Count [%]**	**Count [%]**	**Count [%]**
Gravidity	Prim gravida	24 [11.9]	177 [88.1]	201 [46.3]
	Multigravida	24 [10.3]	209 [89.7]	233 [53.7]
Parity	Primipara	28 [11.8]	209 [88.2]	237 [54.6]
	Multipara	20 [10.2]	177 [89.8]	197 [45.4]
Antenatal follow-up	Complete	17 [4.8]	340 [95.2]	357 [82.3]
	Incomplete	23 [36]	41 [64]	64 [14.75]
	None	8 [61.5]	5 [38.5]	13 [3]
maternal blood group	A	12 [9.8]	111 [90.2]	123 [28.3]
	B	14 [10.6]	118 [89.4]	132 [30.4]
	AB	8 [10.5]	68 [89.5]	76 [17.5]
	O	11 [11.6]	84 [88.4]	95 [22]
	Unknown	3 [37.5]	5 [62.5]	8 [1.8]
RH factor	RH positive	1 [2.6]	38 [97.4]	39 [8.99]
	Rh-negative	44 [11.4]	343 [88.6]	387 [89.2]
	Unknown	3 [37.5]	5 [62.5]	8 [1.8]
Onset of labor	Spontaneous	36 [10.0]	323 [90.0]	359 [82.7]
	Induced	12 [16.0]	63 [84.0]	75 [17.3]
The current mode of delivery	Normal vaginal delivery	28 [10.0]	251 [90.0]	279 [64.3]
	Assisted vaginal delivery	5 [11.1]	40 [88.9]	45 [10.37]
	Cesarean section	15 [13.6]	95 [86.4]	110 [25.4]

### Maternal obstetric conditions

About 15 [10.9%] of neonates born from mothers who had problems of obstetric problems died and 33 [11.1%] of neonates died born from mothers who did not have such problems (Table 3).

**Table 3:** Maternal Gynecologic, and obstetric conditions at NICU, TASH, Addis Ababa, Ethiopia, from 1 January 2015 – 30 December 2021.

**Variables category**		**Outcome [*****N*** = **434]**		
		**Death**	**censored**	
		**Count [%]**	**Count [%]**	**Row total**
Maternal, gynecologic and	Yes	15 [10.9]	123 [89.1]	138 [32]
obstetric problems	No	33 [11.1]	263 [88.9]	296 [68]
Abortion	Yes	4 [8.2]	45 [91.8]	50 [36]
	No	11 [12.4]	78 [87.6]	89 [64]
PROM	Yes	1 [10.0]	9 [90.0]	10 [7.2]
	No	15 [11.0]	113 [89.0]	128 [92.8
Prolonged labor	Yes	2 [5.3]	36 [94.7]	38 [27.5]
	No	13 [13.0]	87 [87.0]	100 [72.5
Previous C/S	Yes	6 [15.0]	34 [85.0]	40 [29]
	No	9 [9.2]	89 [90.8]	98 [71]
Placenta Previa	Yes	3 [18.8]	13 [81.3]	16 [11.6]
	No	12 [9.8]	110 [90.2]	122 [88.4
Oligohydramnios	Yes	2 [14.3]	12 [85.7]	14 [10]
	No	13 [10.5]	111 [89.5]	124 [90]
Chorioaminities	Yes	1 [10.0]	9 [90.0]	10 [7.2]
	No	14 [10.9]	114 [89.1]	128 [92.8
Poly hydramnios	Yes	0 [0.0]	5 [100.0]	5 [3.6]
	No	15 [11.3]	118 [88.7]	133 [96.4
Cephalo-pelvic	Yes	2 [11.8]	15 [88.2]	17 [12.3]
disproportion	No	13 [10.7]	108 [89.3]	121 [87.7
multiple pregnancy	Yes	0 [0.0]	5 [100.0]	5 [3.6]
	No	17 [11.5]	116 [88.5]	133 [96.4

### Maternal medical and related conditions

Neonates born from hypertensive mothers had 18% neonatal death risk than neonates born from non-hypertensive mothers 13.3%. HIV positive mothers had 29% hazard for neonatal death, but HIV negative mothers had 19% hazard of neonatal death (Table 4).

**Table 4:** Maternal medical and related conditions NICU, TASH, Addis Ababa, Ethiopia, from 1 January 2015 – 30 December 2021.

**Variables category**		**Death**	**Censored**	**Row total**
		**Count [%]**	**Count [*N* %]**	**Count [%]**
Maternal medical	Yes	19 [21.3]	70 [78.7]	89 [20.5]
problems	No	29 [8.4]	316 [91.6]	345 [79.5]
Hypertension	Yes	8 [18.2]	26 [81.8]	44 [49.5]
	No	6 [13.3]	49 [86.7]	45 [50.5]
HIV	Yes	7 [29.2]	17 [70.8]	24 [27]
	No	12 [18.5]	53 [81.5]	65 [73]
Diabetes mellitus	Yes	9 [39.1]	14 [60.8]	23 [25.8]
	No	17 [25.8]	49 [74.2]	66 [74.2]
cardiac diseases	Yes	6 [30.0]	14 [70.0]	20 [22.5]
	No	13 [18.8]	56 [81.2]	69 [77.5]
TORCH infections	Yes	1 [9.1]	10 [90.9]	11 [12.4]
	No	18 [23.1]	60 [76.9]	78 [87.6]
Others	Yes	7 [26.9]	19 [73.1]	26 [29]
	No	12 [19.0]	51 [81.0]	63 [71]

### Neonatal medical conditions/assessments

Regarding the neonatal medical conditions; the common medical problems identified were respiratory distress 121 [28%], perinatal natal asphyxia 143 [33%],low APGAR score [<7,194 [44.7%], low birth weight 84 [20%], Prematurity or SGA 88 [21%], sepsis 133 [31%], preterm birth 95 [23%], hypothermia 140 [32.3%], meconium aspiration syndrome 79 [18.2%], neonatal jaundice 177 [40.8%], neural tube defects 31 [7.1%], and trachea esophageal fistula 134 [31%]. Concerning causes of death, PNA 43 [30.1%], sepsis 31 [23.3%], LBW 41 [49%], Respiratory distress 35 [28.9%], low Apgar score 42 [21.6%], SGA 38 [43.2%], preterm 32 [33.7%], hypothermia 12 [8.6%], MAS 3 [3.8%], jaundice 25 [14%], neural tube defects 3 [9.7%] trachesophageal fistula 33 [24.6%] were identified medical conditions related to neonatal death in their NICU stay (Table 5).

**Table 5:** Neonatal medical conditions or assessments who were admitted at NICU of TASH, Addis Ababa, Ethiopia, from 1 January 2015 – 30 December 2021.

**Variable category**		**Outcome[*****N*** = **434]**		**Row total**
		**Death**	**Censored**	
		**Count [%]**	**Count [%]**	**Count [%]**
Maturity of the newborn	AGA	6 [2]	314 [98]	320 [73.7
	LGA	4 [15.4]	22 [84.6]	26 (41)
	SGA	38 [43.2]	50 [56.8]	88 [20.3]
Gestational age	Preterm	32 [33.7]	63 [66.3]	95 [21.9]
	Term	12 [4]	293 [96]	308 [70.9]
	Post-term	4 [11.8]	30 [88.2]	34 [7.8]
Weight at admission	LBW	41 [49]	43 [51]	84 [19.4]
	NBW	6 [2]	335 [98]	341 [78.6
	BIG BABY	1 [12.5]	8 [87.5]	9 [2]
APGAR score at the first minute	≤ 3	24 [22.2]	84 [76]	108 [24.9]
	4–6	21 [9]	213 [91]	234 [53.9]
	≥7	3 [3.3]	89 [96.3]	92 [21.2]
APGAR score at 5 min	≤ 3	28 [37.3]	47 [62.7]	75 [17.3]
	4–6	14 [11.8]	105 [88.2]	119 [27.4]
	≥7	6 [2.5]	234 [87.5]	240 [55.3]
Perinatal asphyxia	Yes	43 [30.1]	100 [69.9]	143 [33]
	No	5 [1.7]	286 [98.3]	291 [67]
Respiratory distress	Yes	35 [28.9]	86 [71.1]	121 [27.9]
	No	13 [4.2]	300 [95.8]	313 [72.1]
Hypothermia	Yes	12 [8.6]	128 [91.4]	140 [32.3]
	No	36 [12.2]	258 [87.8]	294 [76.7]
Neonatal jaundice	Yes	25 [14]	152 [86]	177 [40.8]
	No	23 [8.9]	234 [91.1]	257 [59.2]
Sepsis	Yes	31 [23.3]	102 [76.7]	133 [30.6]
	No	17 [5.6]	284 [9.4]	301 [69.4]
Meconium aspiration syndrome	Yes	3 [3.8]	76 [96.2]	79 [18.2]
	No	45 [12.7]	310 [87.3]	355 [81.8]
Neural tube defects	Yes	3 [9.7]	28 [90.3]	31 [7.1]
	No	45 [11.2]	358 [88.8]	403 [92.9]
Feeding practice	breastfeeding	35 [8.5]	379 [91.5]	414 [95.4]
	Formula feeding	0 (41)	2 [100.0]	2 [0.5]
	Mixed feeding	3 (41)	15 [84]	18 [4.1]
Tracheoesophageal fistula	Yes	33 [24.6]	101 [75.4]	134 [30.9]
	No	15 [5.2]	285 [94.8]	300 [69.1]

### The time to death of a neonate

A total of 434 neonates who were admitted to the NICU of TASH during the study period have been followed from 0 to 28 days. The overall time to death was 17 days [95%CI: 16.71–19.35] with a minimum and the maximum follow up time of 1 and 23 days. In this study, 48 [11.1%] of the neonates died during the follow-up period. Out of 386 censored, 310 [80.3%] were alive and discharged to home, 65 [16.8%] referred and 11 [2.9%] were lost to follow-ups.

**Figure 1:**
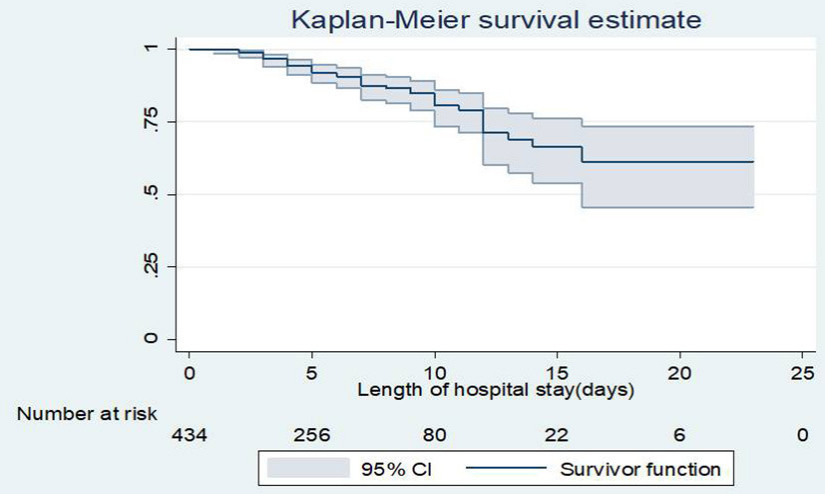
Overall Kaplan-Meier survival estimate of neonates admitted to NICU, TASH, Addis Ababa, Ethiopia, from 1 January 2015 – 30 December 2021.

The overall incidence rate was 19.2 [95% CI: 14.5, 25.03] deaths per 1,000 live births in 2499 person-day observations]. The cumulative proportion of surviving at the end of the1^st^, 7th, 14th, and 23rd days was 99.8, 87.5, 66.3, and 61.2%, respectively. The overall probability of survival of neonate was about 0. 612 [95% CI: 0.46–0.73] for the follow-up period of time (Figure 1).

### Survivorship function between groups of neonates

Those neonates who were diagnosed as small for their gestation had a longer survival time than those who were appropriate for their gestation and large for their gestation 9.8 [95% CI: 8.54–11.06], and the median survival time for small for gestation was 10 [95% CI:7.72–12.2]. The Kaplan-Meier graph curve shows the mean and median survival time of those neonates who had low admission weight was 11 days with [95% CI: 8.82–12.81], and 10 days of median [95% CI: 7.72–12.27], respectively, and for those who had a normal admission weight, their mean survival time was 21 days [19.98–22.10], which was longer than the mean survival time of low birth and big baby. The mean and median survival time of preterm was 10.5 [9.14–11.9], 11 [7.8, 14.23] and for post-term neonates, their mean survival time was 16.8 [12.12–17.3], which was shorter than the mean survival time of term neonates 21 days [19.6–22.2].

### Predictors of neonatal mortality

Results of the multivariable cox proportional hazard regression analysis revealed that neonates who were small for their gestational age were 5 times more likely to die as compared to those who were appropriate for their gestations [AHR:4.8 (95% CI: 2.33–9.72)].

Those neonates who had an APGAR score of less than 7 at 5^th^ min were about 5 times more likely to die as compared to those who had an APGAR of >7 [AHR:5.0 (95% CI: 1.51–15.04)]. Pre-term newborns had 4 times increased hazard of death than term newborns [AHR 4.2 (95% CI: 1.32–8.83)]. Neonates who diagnosed and admitted with perinatal asphyxia were 5 times more likely to die as compared to those who were not asphyxiated [AHR: 5.2 (95% CI: 1.92–14.30)]. Neonates born from mothers who did not attend ANC visits during their pregnancy were four times at higher risk of death than neonates born from mothers who had antenatal follow up [AHR: 3.7 (95% CI: 1.86,7.60)]. Low birth weight neonates were 7.3 times more likely to die than normal birth weight neonates [AHR: 7.3 (95% CI: 2.69, 19.91)]. Neonates who were in respiratory distress had nearly 2.5 times more likely to die than those who were not in distress [AHR: 2.5 (95% CI: 1.24–5.09)]. Those who had neonatal sepsis at the time of admission were three times more likely to die than those who did not have sepsis [AHR: 3.4 (95% CI: 1.71–4.01)]. Neonates diagnosed as having TEF had 2.2 times more Hazardous than those neonates who did not have [AHR: 2.2 (95% CI: 1.13–4.32)] (Table 6).

**Table 6:** Results of the bivariable and multivariable Cox regression on predictors of neonatal mortality in TASH, Addis Ababa, Ethiopia, 1 January 2015 – 30 December 2021.

**Variables**	**Category**	**Outcome**		**CHR [95% CI]**	**AHR [95% CI]**
		**Died**	**Censored**		
Gravidity	Primgravida	24 [11.9]	177 [88.1]	0.63 [0.36,1.2]	1.7 [0.61–4.5]
	Multigravida	24 [10.3]	209 [89.7]	–	–
Antenatal follow-up	Complete	17 [4.8]	340 [95.2]	–	–
	Incomplete	23 [36]	41 [64]	8.5 [4.5, 16.02]	3.7 [1.86, 7.60]^**^
	None	8 [61.5]	5 [38.5]	9.4 [4.0–22.0]	4.0 [1.41–10.71]^*^
Rh factor	Positive	1 [2.6]	38 [97.4]	0.3 [0.04,2.12	0.8 [0.02–17.33]
	Negative	44 [11.4]	343 [88.6]	–	–
	UK	3 [37.5]	5 [62.5]	1.93 [0.6–6.3]	–
Maturity of the newborn	AGA	6 [2]	314 [98]	–	
	LGA	4 [15.4]	22 [84.6]	9 [2.5–32.9]	2.5 [1.32–14.21]^*^
	SGA	38 [43.2]	50 [56.8]	20 [8.7–49.3]	4.8 [2.33,9.72]^*^
Gestation age	Preterm	32 [33.7]	63 [66.3]	8.2 [4.2–16]	4.2 [1.32–8.83]^*^
	Term	12 [4]	293 [96]		
	Post term	4 [11.8]	30 [88.2]	2.7 [0.86–8.3]	0.6 [0.07–5.44]
Weight	LBW	41 [49]	43 [51]	21 [9.03,50.34]	7.3 [2.69,19.95]^*^
	NBW	6 [2]	335 [98]	–	–
	BIG BABY	1 [12.5]	8 [87.5]	10.6 [1.3, 49]	3.0 [1.23–5.12]^*^
APGAR score at the first minute	≤ 3	24 [22.2]	84 [76]	3.9 [1.15–12.7]	3.0 [0.54–10.04]
	4–6	21 [9]	213 [91]	2.7 [0.8–9]	2.4 [0.61–12.03]
	≥7	3 [3.3]	89 [96.3]	–	–
APGAR score at 5 min	≤ 3	28 [37.3]	47 [62.7]	14 [5.8–33.7]	5.0 [1.51–15.04]^**^
	4–6	14 [11.8]	105 [88.2]	2.2 [0.8–5.8]	2.0 [0.42–12.02]
	≥7	6 [2.5]	234 [87.5]	–	–
Perinatal asphyxia	Yes	43 [30.1]	100 [69.9]	0.06 [0.03,0.2]	5.2 [1.92–14.30]^**^
	No	5 [1.7]	286 [98.3]	–	–
Respiratory distress	Yes	35 [28.9	86 [71.1]	0.12 [0.08–0.3]	2.5 [1.24– 5.09]^*^
	No	13 [4.2]	300 [95.8	–	–
Jaundice	Yes	25 [14]	152 [86]	0.6 [0.3,1.009]	0.9 [0.26–3.13]
	No	23 [8.9]	234 [91.1]	–	
Sepsis	Yes	31 [23.3]	102 [76.7]	0.2 [0.13–0.4]	3.4 [1.71–4.01]^**^
	No	17 [5.6]	284 [9.4]	–	
TEF	Yes	33 [24.6]	101 [75.4]	0.2 [0.01–0.3]	2.2 [1.13–4.32] ^*^
	No	15 [5.2]	285 [94.8]		

** *p < 0.001*,

* *p < 0.05*.

## Discussion

The study showed that the overall incidence rate was 19.2 deaths per 1,000 live births. Neonates were followed for 2,499 [95 CI; 2,326, 2,671] days, resulting in an overall incidence rate of 19.2 deaths per 1,000 live births. This finding was lower EDHS 2019 report [33 deaths per 1000 live births], respectively (24), Tigray region 62.5 per 1,000 live births (25), Wolayita Sodo teaching and university hospital 27 per 1,000 live births (26), and Sidama Zone in South nation 41 per 1,000 live births (27). This difference could be due to sample size taken, study design employed, study period, and also might be related to the variation of the health service coverage. Besides, this tertiary hospital gives service to those neonates who were referred from different areas of the country for better management. This made the referral system linked to treat to those client who couldn‘t‘ treated in other hospitals. Hence, the report from this hospital is lower than EDHS report. Because, EDHS data included reports from different institution from district hospital to tertiary hospital.

The result of this study showed that about 11.1% newborns were died, which was less than studies done in Cameroon 15.7% (28). The difference could be sources of data; this study reviewed data from only NICU admitted neonates, and might be also related to the quality of health care service given.

In this study, about 70% neonatal deaths were registered in the early neonatal period [0–6 days], and 30 % of were died during the late neonatal period which was inconsistent with study done in Kara Mara, in which 96 % were early neonatal deaths (29) and a study done in NICU at Arba Minch General Hospital, Southern Ethiopia 91% (30). This variation could be explained in terms of variations in the presence of obstetric complications during pregnancy, and failure in early identification and poor management of maternal medical, obstetric and gynecological complications, which determines mortality in the first week of neonatal life. Moreover, this difference perhaps due to study period difference along with advance in the health care system that people's attitudes and awareness about health conditions that bring the neonates for ill health and increase in health-seeking behavior.

In this study, being pre-term newborn had an increased hazard of death than term neonates and was consistent with studies done in Cameroon (28), and Ghana (31) This might be due to preterm babies are not fully prepared to live outside the uterus, as their bodies are not fully matured than full-term babies. In addition, this variation may be due to study period, variation in sample size taken and study design conducted.

The finding of this study had a lesser hazard of death than studies done in Ghana (32), and a pooled analysis was done on low income and middle-income countries (33), this variation might be due to methodological difference, and presence of less maternal predisposing factors for preterm birth in this study area.

Another significant predictor of neonatal death was perinatal asphyxia. Asphyxiated neonates were more likely to die than neonates without asphyxia, which makes it similar to a study done in NICU of Komfo Anokye Teaching Hospital [KATH] in Ghana (34), and NICU of Gondar university hospital (22). This might be related to asphyxiated neonates failed to establish breathing at birth than non-asphyxiated neonates, but this study finding was different from a study done in Wolayta Sodo about 2 times (26). This might be due to early diagnosis and treatment in Wolayeta Sodo before complications occur.

In this study, an APGAR score of <3 was found to be a significant factor, which was similar with the previous studies done in Cameroon (28), EasternEthiopia (35), Kenya (36), China (32, 37), Swedn (38) and Arba Minch (30) and Eastern. This might be related to 5^th^ min APGAR score which is a useful index of the response to resuscitation efforts, and low APGAR score neonates are risky to be oxygen-deprived than an APGAR of >7. This discrepancy might be explained by the sample size, and obstetrical care, and neonatal resuscitation efforts. Low birth weight neonates were more likely to die than normal birth weight neonates, this was in line with a study done in NICU of Kath in Eastern Ethiopia (35), and in Northern Ethiopia (25). This might be due to a baby with low birth weight is at increased risk for complications of low birth asphyxia, hypothermia, feeding problem, infections, and prematurity, which might increase their risk of death than normal birth weight babies.

Those neonates who were small for their gestational age were more likely to die than those neonates who had appropriate gestational age, which was in line with a study done in Canada (34). Those who had neonatal sepsis at the time of admission were more hazardous to die than those who did not have sepsis, a consistent finding was observed in a study conducted at NICU of Gondar referral hospital (22), Tigray Hospitals (39) and Kenya (36). Neonates admitted with a diagnosis of respiratory distress were more prone to death than those who were not in distress, and was consistent with a study done in Wolayta Sodo (26), but this study had a lesser hazard than a study done in Ghana (32). This study illustrated that about 29% of neonatal death was related to respiratory distress, which was more hazardous than a study done in Kara Mara, Somali region of Ethiopia 24.4% (29). However, it was consistent with studies in Tigray (39), and Gondar (22).

Trachea esophageal fistula (TEF) was the other predictor of neonatal death. Neonates diagnosed as having TEF were more hazardous than those neonates who did not have. In this study, about 24.6% of neonates were died due to diagnosed TEF, which is consistent with previous study (40). Neonates with this condition potentially develop aspiration and faced feeding difficulties resulting in low calories and immunity which affect their growth and development. Neonates could easily dehydrate due to decreased fluid intake, inadequate breastfeeding.

### Limitations and strength of the study

Strength of this study includes:- This is an approach to clearly indicate the temporal sequence of the predictor and outcome variable (death); Data reviewing was conducted by trained nurses, and standardized data abstraction tool was used and It enables to estimate the survival time of neonates, and identify predictors for future trend estimation and other related prevention and intervention programs.

Using secondary sources may result in loss of potential predictors, which may not be addressed in this study design like; maternal nutritional status, educational level, and family-related issues. As the incomplete charts were excluded from the study there may be selection bias. This study was done retrospectively which might cause recall bias due to failure of the caregiver to remember what was happened previously.

## Conclusion

The overall incidence rate during the 2,499 person-time was 19.2 deaths per 1,000 person-day observation with a time to death of 17 days.

The independent predictors were incomplete or no maternal antenatal follow up, being small for gestation, preterm, low birth weight, low 5^th^ min Apgar score, neonatal sepsis, respiratory distress, perinatal asphyxia, and TEF. This recommended that maternal health during pregnancy, including quality of antenatal care utilization should be further strengthened, and risky pregnant mothers should be identified antenatally. Initiating different strategies to work on early identification, quality and continuous care for neonates coming with an assessment of preterm, low birth weight, perinatal asphyxia, respiratory distress, and sepsis and tracheoesophageal fistula and implementing it to ensure quality neonatal care.

## Data availability statement

The original contributions presented in the study are included in the article/Supplementary material, further inquiries can be directed to the corresponding author.

## Ethics statement

The studies involving human participants were reviewed and approved by AAU, College of Health Science, School of Nursing and Midwifery Institutional Review Board. Written informed consent to participate in this study was provided by the participants' legal guardian/next of kin.

## Author contributions

MM, GM, WK, FT, and BA was responsible to conception, design, acquisition of data, analysis and interpretation of data, and was responsible in the writing the original research and preparation of the manuscript for publication. BG and KD were responsible for design, supervision, and reviewing it for accuracy and integrity. All authors play roles in this research article, read, and approved the final manuscript.

## Conflict of interest

The authors declare that the research was conducted in the absence of any commercial or financial relationships that could be construed as a potential conflict of interest. Reviewers WS and YA, declared a shared affiliation with the authors to the handling editor at the time of review.

## Publisher's note

All claims expressed in this article are solely those of the authors and do not necessarily represent those of their affiliated organizations, or those of the publisher, the editors and the reviewers. Any product that may be evaluated in this article, or claim that may be made by its manufacturer, is not guaranteed or endorsed by the publisher.

## Abbreviations

AHR: Adjusted Hazard Ratio
AIDS: Acquired Immune Deficiency Syndrome
ANC: Anti Natal Care
AOR: Adjusted Odds Ratio
APGAR, Appearance, Pulse, Grimily, Activity: and Respiration
CSA: Central Statistical Agency
EBF: Exclusive Breast Feeding
EDHS: Ethiopian Demographic and Health Survey
EONS: Early Onset Neonatal Sepsis
FMOH: Federal Ministry of Health
GA: Gestational Age
GHO: Global Health Observatory
HBsAg: Hepatitis B Surface Antigen
HMD: Hyaline Membrane Diseases
HR: Hazard Ratio
LBW: Low Birth Weight
NICU: Neonatal Intensive Care Unit
NMR: Neonatal Mortality Rate
RDS: Respiratory Distress Syndrome
SDG: Sustainable Development Goals
TASH: Tikur Anbessa Specialized Hospital
UNICEF: United Nations International Children Emergency Fund.
