# Supplementary material for: Time to death and its predictors among neonates who were admitted to the neonatal intensive care unit at tertiary hospital, Addis Ababa, Ethiopia: Retrospective follow up study
Source: Front Pediatr. 2022 Aug 29;10:913583. doi: 10.3389/fped.2022.913583 (PMC9476827; doi:10.3389/fped.2022.913583)
Supplement: Supplementary file 1 [file Data_Sheet_1.DOCX]

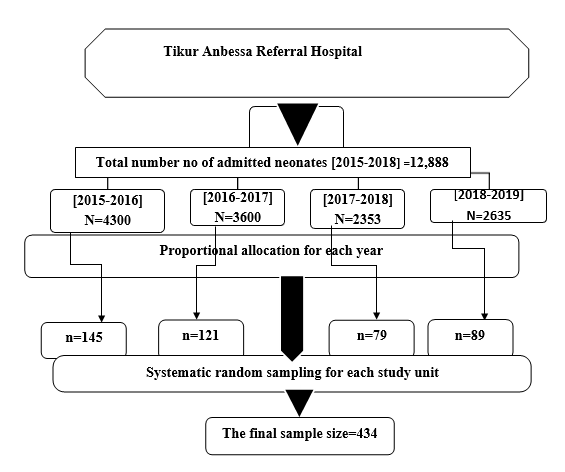


### suppl 1: Schematic presentation of sampling procedure to assess Survival status and predictors of mortality among neonate admitted to NICU from January 1st, 2015 – December 30th in Tikur Anbessa Specialized Hospital, Addis Ababa, Ethiopia, 2021.
